# Phenotypic Parallelism during Experimental Adaptation of a Free-Living Bacterium to the Zebrafish Gut

**DOI:** 10.1128/mBio.01519-20

**Published:** 2020-08-18

**Authors:** Jarrett F. Lebov, Brandon H. Schlomann, Catherine D. Robinson, Brendan J. M. Bohannan

**Affiliations:** a Institute of Ecology and Evolution, University of Oregon, Eugene, Oregon, USA; b Institute of Molecular Biology, University of Oregon, Eugene, Oregon, USA; c Department of Physics, Materials Science Institute, University of Oregon, Eugene, Oregon, USA; d Institute for Genome Sciences, University of Maryland, Baltimore, Maryland, USA; Brigham and Women’s Hospital/Harvard Medical School

**Keywords:** bacteriology, biofilm, ecology, evolution, genetics, genomics, host-microbe, *Shewanella*, swimming motility, symbiosis, zebrafish

## Abstract

Although animals encounter a plethora of bacterial species throughout their lives, only a subset colonize vertebrate digestive tracts, and these bacteria can profoundly influence the health and development of their animal hosts. However, our understanding of how bacteria initiate symbioses with animal hosts remains underexplored, and this process is central to the assembly and function of gut bacterial communities. Therefore, we used experimental evolution to study a free-living bacterium as it adapts to a novel vertebrate host by serially passaging replicate populations of Shewanella oneidensis through the intestines of larval zebrafish (Danio rerio). After approximately 200 bacterial generations, isolates from evolved populations improved their ability to colonize larval zebrafish during competition against their unpassaged ancestor. Genome sequencing revealed unique sets of mutations in the two evolved isolates exhibiting the highest mean competitive fitness. One isolate exhibited increased swimming motility and decreased biofilm formation compared to the ancestor, and we identified a missense mutation in the mannose-sensitive hemagglutinin pilus operon that is sufficient to increase fitness and reproduce these phenotypes. The second isolate exhibited enhanced swimming motility but unchanged biofilm formation, and here the genetic basis for adaptation is less clear. These parallel enhancements in motility and fitness resemble the behavior of a closely related *Shewanella* strain previously isolated from larval zebrafish and suggest phenotypic convergence with this isolate. Our results demonstrate that adaptation to the zebrafish gut is complex, with multiple evolutionary pathways capable of improving colonization, but that motility plays an important role during the onset of host association.

## Abstract (precis)

Although animals encounter many bacterial species throughout their lives, only a subset colonize vertebrate digestive tracts, and these bacteria can profoundly influence the health and development of their animal hosts. We used experimental evolution to study a free-living bacterium as it adapts to a novel vertebrate host by serially passaging replicate populations of Shewanella oneidensis through the intestines of larval zebrafish (Danio rerio). Our results demonstrate that adaptation to the zebrafish gut is complex, with multiple evolutionary pathways capable of improving colonization, but that motility plays an important role during the onset of host association.

## INTRODUCTION

Bacterial lineages have radiated into practically every imaginable niche on Earth (1, 2). In particular, the vertebrate digestive tract houses bacterial communities whose composition is distinct from those found in surrounding environments (3, 4), and this suggests that host-associated bacteria maintain certain traits that enable them to colonize animal hosts. In order to establish and maintain host-association, bacteria must surmount a multitude of complex challenges, including traversing diverse physical landscapes, harvesting energy from dynamic nutrient sources, and protecting themselves from antimicrobial compounds. Thus, the number of traits involved in host-association is likely enormous (5, 6). Despite this complexity, previous analyses indicate that novel host-microbe symbioses have arisen multiple times throughout evolutionary history (7). However, it is unknown which suites of traits enable bacteria to transition to host association or how likely they are to evolve.

It is well established that bacteria residing in vertebrate digestive tracts have substantial impacts on the health and development of their animal hosts (8–11). Consequently, many researchers have sought to understand which traits provide bacteria the capacity to colonize the vertebrate gut (12–17), but this body of work has relied almost exclusively on snapshots of host-microbe relationships after they have evolved. Because information may be lost over the course of evolution, it is difficult to infer traits that facilitate transitions to host association by limiting examinations solely to strains that have already made such a transition (18).

To better understand how bacteria initiate host associations, we took an experimental evolution approach involving the serial passage of a free-living bacterial strain through the digestive tracts of a model vertebrate. When combined with genomic sequencing of evolving lineages, this strategy enables the observation of evolutionary changes in genotype at fine temporal scales (19). Phenotypic and fitness assays can then be performed, and this information can be synthesized to understand which sets of traits facilitate adaptation, how these traits interact to improve fitness, and in what order these traits are likely to evolve (20–23).

Many insights have been gained from employing this type of approach *in vitro*, and experimental evolution practitioners have begun expanding into *in vivo* environments to investigate evolutionary dynamics within hosts (24–28). A recent example of this was conducted by Robinson et al., who experimentally adapted an *Aeromonas* strain previously isolated from zebrafish (Danio rerio) to germfree (GF) larval zebrafish to explore how this bacterium might increase its association with its vertebrate host (29). These researchers found that evolved *Aeromonas* isolates achieved higher relative abundances in the digestive tracts of larvae during competition against their ancestral strain. Further, these increases in relative fitness appeared to be explained by augmented motility of evolved isolates, leading to higher rates of colonization.

**FIG 1:**
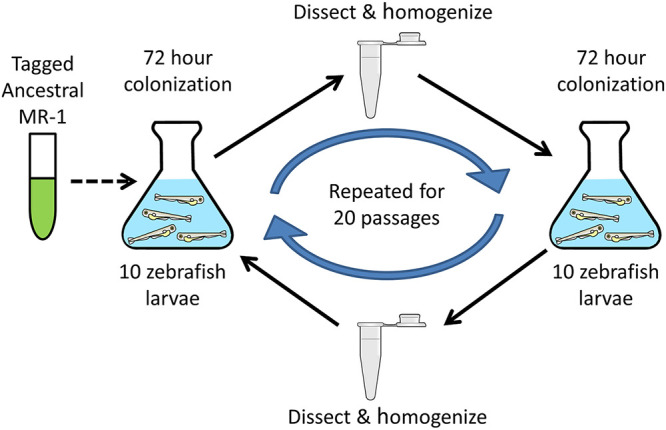
Serial passage schematic. GF larvae were incubated with MR-1 populations for 72 h, and then 10 larval guts were dissected and homogenized. A sample of the homogenate was used to inoculate a subsequent set of GF larvae. This cycle was repeated for 20 passages. The dotted arrow indicates that no additional S. oneidensis ancestor was added to the experimental system after the first passage.

For their study, Robinson et al. used a bacterial symbiont that had been isolated from a zebrafish gut, and thus it is not known how this *Aeromonas* species’ relationship with zebrafish originated or which traits may have been involved in the initiation of this process. Therefore, while this previous work investigated how established bacterial symbionts can improve their ability to colonize the host, we sought to understand how a bacterium might initiate a novel host-microbe symbiosis. We accomplished this by serially passaging a bacterial species with no documented history of an association with a metazoan host, Shewanella oneidensis MR-1, through the digestive tracts of a model vertebrate, zebrafish (Fig. 1). We chose this *Shewanella* strain because it is one of the best-studied bacterial strains isolated from a non-host environment, it is genetically tractable (30), and it is closely related to a *Shewanella* strain known to colonize larval zebrafish. We predicted these attributes would maximize our ability to map evolved phenotypes to genotypes.

After 20 passages, we observed that evolved isolates from five of the six replicate lines demonstrated a significantly improved ability to colonize larvae compared to their unpassaged ancestor. We then characterized the two evolved isolates with the highest average relative fitness and discovered that each isolate had accumulated a distinct set of mutations. Interestingly, despite these different mutation profiles, both isolates evolved augmented swimming motility relative to the ancestral reference strain, demonstrating phenotypic parallelism in the adaptive trajectories of these two independently evolved isolates. Our findings show that bacteria can rapidly evolve novel host associations and suggest that swimming motility is advantageous for colonizing aquatic hosts.

## RESULTS

### MR-1 colonizes zebrafish at lower densities than a closely related *Shewanella* zebrafish isolate.

**FIG 2:**
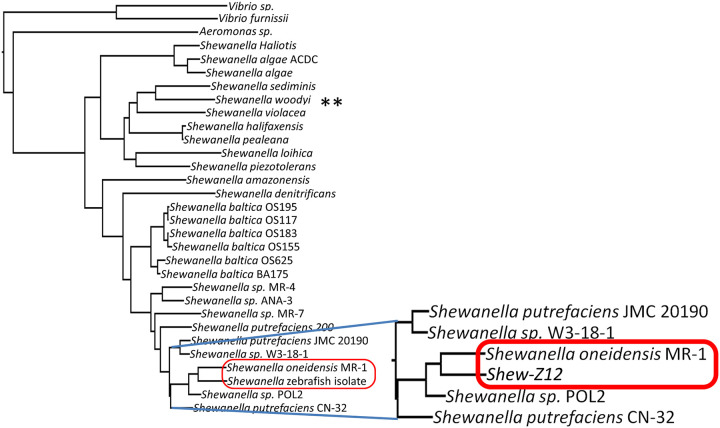
Relatedness of MR-1 to other *Shewanella* species. A phylogenetic tree, based on 16S gene sequences (Table S2), shows the relationship between multiple different *Shewanella* species.

Gut-associated bacteria are routinely isolated from their animal hosts. Although MR-1 has never been found within a host gut, other strains belonging to the *Shewanella* genus are common in the larval zebrafish microbiota (31). Indeed MR-1 shares a recent common ancestry with a *Shewanella* species that was isolated from the zebrafish gut (Fig. 2; *Shewanella* ZOR0012 [referred to here as Shew-Z12]). Interestingly, a whole-genome comparison revealed that MR-1 shares an average nucleotide identity (ANI) of approximately 89% with Shew-Z12, compared to 72% with the more distantly related Shewanella woodyi (Fig. 2). The high degree of overlap between MR-1 and Shew-Z12 was further reflected when we compared the protein sequence alignments of Shew-Z12 or *S. woodyi* against our MR-1 reference genome (see Fig. S1 in the supplemental material). Relative to *S. woodyi*, MR-1 displays much higher levels of amino acid sequence identity with Shew-Z12 on a per-gene basis, implying greater amounts of functional conservation between MR-1 and Shew-Z12 (see Fig. S1).

10.1128/mBio.01519-20.1 FIG S1 Whole-genome amino acid comparisons per gene. Whole-genome alignments of Shew-Z12 (outer ring) or S. woodyi (inner ring) amino acid sequences against the MR-1 reference genome are shown on a per-gene basis. Color shading around each ring indicates sequence identity (key at the top of the figure). Alignments were conducted using the SEED Viewer v2.0 sequenced-based comparison tool (77). Download FIG S1, TIF file, 0.8 MB. Copyright © 2020 Lebov et al. 2020 Lebov et al. This content is distributed under the terms of the Creative Commons Attribution 4.0 International license.

**FIG 3:**
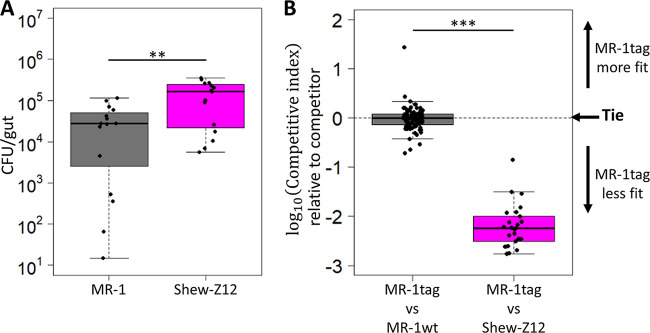
Fitness comparison between MR-1 and Shew-Z12. (A) Colonization density achieved in larval guts after 72 h of colonization under monoassociation conditions for indicated strains. Dissected guts were plated on TSA and CFU were counted. Each point represents a single dissected gut. (B) Competitive ability of unpassaged fluorescently tagged MR-1 strain competed against an untagged version of itself (MR-1wt; left column) or an untagged Shew-Z12 (right column). Each point represents the competitive index measured for a single larval gut (see Materials and Methods for details on how competitive indices were calculated).

Given the close phylogenetic relationship between MR-1 and Shew-Z12, we wanted to determine whether MR-1 and Shew-Z12 would have similar larval gut colonization characteristics. To assess this, we compared the ability of each strain to colonize GF larval zebrafish guts under both monoassociation and competitive conditions. In both scenarios, flasks containing 10 to 15 GF larval zebrafish were inoculated with bacterial densities of ∼10^3^ CFU/ml. Gut colonization was assessed by dissecting and plating larval digestive tracts after 72 h of exposure. In monoassociation MR-1 colonized GF larvae at ∼10-fold lower densities than Shew-Z12 (Fig. 3A). To assess the competitive fitness of these strains, we competed a strain of MR-1 tagged with a neutral fluorescent marker against Shew-Z12 at a one-to-one ratio and quantified relative fitness using a competitive index. The competitive index was calculated by dividing the ratio of each competitor observed in dissected guts by the ratio of each competitor in the inoculum. We found that MR-1 significantly underperformed the Shew-Z12 isolate and that this difference was greater than could be explained by the difference in abundances in monoassociation (Fig. 3B). Given that MR-1 was neither able to colonize larvae to the same capacity as Shew-Z12 in monoassociation, nor able to compete effectively with this closely related host isolate, we concluded that MR-1 has the potential to improve this host association. We speculated that, given its recent divergence from Shew-Z12, adapting MR-1 to the host from which Shew-Z12 was isolated could provide some insight into the adaptive pathways available to bacteria as they transition to life as a host-associated symbiont.

### Serial passage increased fitness in the gut.

**FIG 4:**
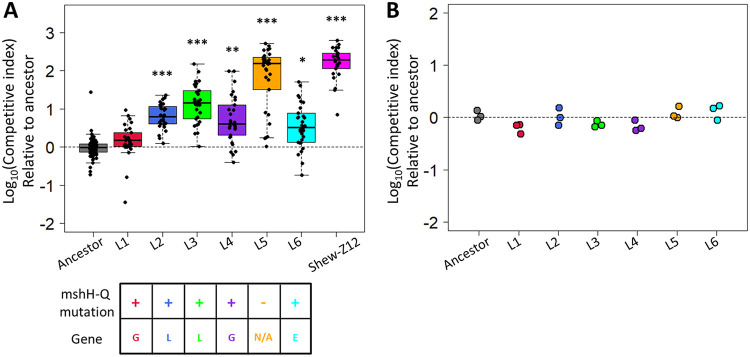
Competitive fitness of evolved S. oneidensis isolates. (A) Competitive ability of MR-1 isolates from each replicate evolved population against the ancestral MR-1 reference strain. An ancestral competition between tagged and untagged ancestors (leftmost box) is shown as a control to show the neutrality of the fluorescent tags, and these data are identical to the leftmost box in Fig. 3B. A linear model was used to compare each group against the ancestral control (*, *P* < 0.05; **, *P* < 0.01; ***, *P* < 0.001). Each point represents the competitive index measured for a single larval gut culled from at least three replicate flasks (*n* = 89 guts for ancestor versus ancestor, 25 guts for Shew-Z12 versus ancestor, and 30 guts for all other groups.). The grid at the bottom indicates whether each evolved isolate contained a mutation in the *msh* operon. (Top row) A “+” indicates the presence of a *msh* operon mutation, and a “–” indicates the absence of a *msh* operon mutation. The bottom row indicates which gene *msh* operon mutations could be found within. (B) Competitive ability of each replicate evolved MR-1 population competing against the ancestral MR-1 reference strain in TSB. Each point represents the competitive index measured for a single biological replicate.

To understand how MR-1 would adapt to a vertebrate host gut, we serially passaged six replicate populations through the digestive tracts of GF larval zebrafish (Fig. 1A). At the start of the experiment, each population was composed of two fluorescently marked MR-1 isolates (dTomato, MR-1dT; gfp, MR-1gfp) so that passaged populations could be distinguished from the unpassaged ancestor, and adaptive events could be inferred from changes in each tag’s frequency within the evolving populations (see Fig. S2). After 20 passages, we assayed the fitness of a single, randomly selected isolate per evolved population by competing each isolate against its unpassaged ancestor. Each competition was performed as described above for the MR-1 versus Shew-Z12 competitions. Of the isolates we tested, five of the six outcompeted the ancestral strain, exhibiting competitive indices that were significantly different from the ancestral control group (Fig. 4A). These improvements were not likely due to adaptation to the general lab environment, because competitions between replicate evolved populations and the MR-1 ancestor in rich media (tryptic soy broth) produced competitive indices not significantly different from 1.0 (Fig. 4B).

10.1128/mBio.01519-20.2 FIG S2 Representation of the dTomato fluorescent tag during serial passage. The proportion of dTomato-tagged MR-1 CFUs counted for each serial passaged population after each passage is shown. Download FIG S2, TIF file, 0.2 MB. Copyright © 2020 Lebov et al. 2020 Lebov et al. This content is distributed under the terms of the Creative Commons Attribution 4.0 International license.

### Comparative genomics revealed multiple candidate adaptive mutations.

To investigate the genetic determinants of the adaptation, we sequenced the genomes of each passage 20 evolved isolate. The reads from each isolate were aligned to the ancestral reference genome to identify mutations that have accumulated during serial passage. This analysis revealed numerous mutations per genome (see Table S1 in the supplemental material), which made it difficult to distinguish adaptive mutations from nonadaptive mutations. For example, mutations with neutral or even slightly deleterious impacts on fitness could have increased in frequency simply through their linkage with another, more beneficial mutation.

10.1128/mBio.01519-20.4 TABLE S1 Candidate adaptive mutations. Mutations are listed for four isolates per evolved population. Each isolate is color-coded by the fluorescent tag it contained (red, dTomato; green, gfp). Except for mshH-Q annotations (annotated with RAST v2.0), gene annotations are based on Prokka v1.12 (Seemann [67]). Color-coding for gene descriptions: green, synonymous mutation; blue, missense mutation; red, nonsense mutation; black, all other mutations. Yellow-shaded cells indicate mshH-Q mutations. Total number of mutations: L1, 3; L2, 3; L3, 17; L4, 6; L5, 4; L6, 11. Download Table S1, XLSX file, 0.04 MB. Copyright © 2020 Lebov et al. 2020 Lebov et al. This content is distributed under the terms of the Creative Commons Attribution 4.0 International license.

10.1128/mBio.01519-20.5 TABLE S2 Metadata for 16S genes used to create the phylogenetic tree featured in Fig. 2. Download Table S2, XLSX file, 0.02 MB. Copyright © 2020 Lebov et al. 2020 Lebov et al. This content is distributed under the terms of the Creative Commons Attribution 4.0 International license.

Therefore, to infer adaptive mutations, we focused on mutations that had accumulated in similar genomic regions across several of our evolved isolates, as such events would be unlikely to occur by chance. In five of the six evolved isolates, we observed mutations in the mannose-sensitive hemagglutinin pilus operon (the operon containing *mshH* to *mshQ* [*mshH-Q*]; Fig. 4A; see also Table S1, yellow shaded) suggesting that the Msh pilus encoded by this operon likely influences larval gut colonization. These mutations were located in the *mshG* (L1 and L4), *mshL* (L2 and L3), and *mshE* (L6) genes (Fig. 4A; see also Table S1), which encode integral membrane platform, secretin, and ATPase proteins, respectively (32). Interestingly, the Msh pilus has been shown to play a role in a number of other host-microbe systems. For instance, within the *Vibrio* genus, Msh pili have been suggested to be important for adherence to human intestinal cells (33), as well as colonization of the digestive tract of Caenorhabditis elegans (34), and the light organ of *Euprymna tasmanica* (35). In addition, in both *Vibrio* and *Pseudomonas* systems, evidence suggests this pilus can interact with components of the mammalian immune system (12, 36). These observations strengthen the hypothesis that MR-1’s Msh pilus was a target of selection in our study.

Notably, the L1 isolate contained a mutation in the *mshG* gene (similar to L4) but did not have a significant colonization advantage over the ancestor (see Table S1). If the other two mutations in L1 were deleterious and recently acquired, it could reconcile L1’s nonadaptive performance with its presence in its population after 20 passages. Alternatively, if the specific *mshG* mutation observed in L1 and L4 is not advantageous, the difference in relative fitness exhibited by these two isolates might be explained by the additional mutations that are unique to each isolate. More detailed evolutionary genomic analyses that clarify the chronology of accumulated mutations in our evolved isolates could help to establish the basis for L1’s apparent lack of adaptation.

**FIG 5:**
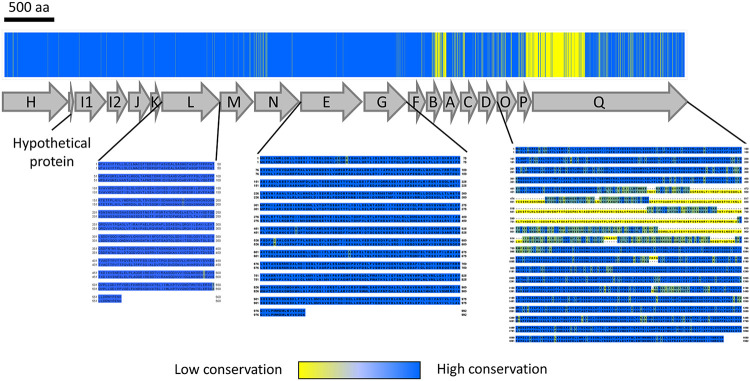
MshOP amino acid conservation between MR-1 and Shew-Z12. The bar across the top shows the MshH-Q amino acid conservation between MR-1 and Shew-Z12. A schematic depicting the organization of the *mshH-Q* operon is shown below. Per site, blue indicates the same amino acid is present in both species, while yellow indicates mismatches. Aligned sequences are shown for genes in which we observed mutations in our evolved isolates. In each row of the displayed sequences, MR-1 is featured on top and Shew-Z12 is featured on the bottom. MshQ did not accumulate mutations in our experiment, but it is shown to illustrate that much of the divergence in this protein resulted from a difference in length between MR-1 and Shew-Z12. The color coding in each sequence alignment indicates the degree of conservation. Amino acids with similar biochemistry are bluer, while those with divergent biochemistry are more yellow. A scale bar above the figure indicates a length of 500 amino acids.

To assess whether the *msh* operon mutations, we observed provided evidence that MR-1 was on a similar adaptive trajectory to one potentially taken by Shew-Z12, we compared the proteins encoded by the *msh* operon (*mshH-Q*) of our reference MR-1 strain to that of Shew-Z12. If evolved MR-1 isolates were on a convergent adaptive trajectory with Shew-Z12, we expected that the mutations found in our evolved isolates would result in amino acid residues similar in identity or biochemistry to those found in Shew-Z12. However, based on our *msh* operon protein alignment we did not find any evidence that differences between the MR-1 and Shew-Z12 were ameliorated by evolved mutations. Our alignment did reveal several regions with elevated divergence between ancestral MR-1 and Shew-Z12 (Fig. 5). In particular, Shew-Z12’s MshQ protein appeared to be noticeably larger than MR-1’s MshQ protein. The fact that there were substantial regions of divergence between the MshH-Q amino acid sequences of MR-1 and Shew-Z12 left open the possibility that the adaptive changes we observed might amount to evolutionary convergence at the functional or regulatory level. Determining this definitively would require more mechanistic and expression-focused approaches, respectively.

The L5 isolate, which exhibited the highest mean fitness of the isolates we tested, contained an entirely unique set of mutations that were not observed in any other evolved isolates. This raises the potential that the L5 isolate may be on a divergent adaptive trajectory, evolving unique phenotypes associated with the exploitation of a distinct niche. Alternatively, it is also possible that L5 may be on a parallel phenotypic evolutionary trajectory, evolving phenotypes similar to those of the other evolved isolates, although via a different set of mutations. To distinguish between these hypotheses, we examined the phenotypes of aggregation behavior and motility in isolate L5 and in isolate L3, the Msh-mutant-containing isolate with the highest mean fitness (Fig. 4).

Several recent studies have demonstrated that aggregation and motility behaviors can have deterministic impacts on the spatial distribution and competitive dynamics of bacteria within the larval zebrafish gut (37–39), and aggregative behaviors are thought to be a crucial step during successful infection by several human pathogens (33, 40, 41). In addition, shifts in motility could impact the rate with which our evolved isolates encounter larval hosts or navigate to their optimal habitat within the gut (14). Therefore, changes in either of these phenotypes could alter how evolved isolates compete with the ancestral MR-1 strain, and differences in these phenotypes between the L3 and L5 isolates could suggest they have adapted to separate niches within our host system.

### L3 and L5 isolates exhibit different biofilm phenotypes.

**FIG 6:**
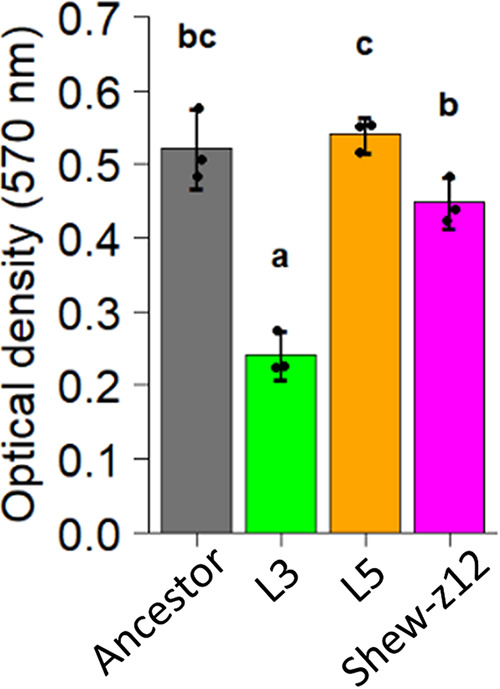
Crystal violet biofilm assay comparing L3, L5, and *Shewanella* sp. strain ZOR0012 to the ancestor. The optical density (570 nm) corresponds to the crystal violet intensity. Higher optical density readings indicate more robust biofilms. Statistical groupings are indicated by letters above each bar for a significance threshold of *P* < 0.05. Letters in common between groups indicate the absence of a significant difference in each group’s mean. Only the L3 isolate shows a significantly altered biofilm phenotype compared to the wild-type ancestor.

We compared the capacity of each isolate to form biofilms using static polystyrene plate-based biofilm assays in larva conditioned medium (LCM). This medium was collected from flasks of GF larval zebrafish at 4 days postfertilization (dpf)—the time when we inoculate larvae during competition assays—and it should provide a similar nutrient profile to that experienced by MR-1 during our evolution experiment. We found that L5’s biofilm phenotype was comparable to that of the ancestor, while L3 had a reduced biofilm phenotype (Fig. 6). We also assessed how the biofilm phenotypes of the L3 and L5 isolates compared to that of Shew-Z12 and found that Shew-Z12 had a mean biofilm phenotype that was intermediate between L3 and L5, although not significantly different from the MR-1 ancestral strain (Fig. 6). Given that L3, L5, and Shew-Z12 are all capable of outcompeting the MR-1 ancestor in their ability to colonize GF larval zebrafish guts, and yet these strains display a range of biofilm phenotypes under the conditions tested, we conclude that our biofilm assay is not capable of predicting competitive fitness *in vivo*. However, our results demonstrate that the unique mutational profiles of the L3 and L5 isolates result in distinct aggregative behaviors.

### L3 and L5 are more motile than the ancestor.

Since Robinson et al. previously demonstrated that serially passaged *Aeromonas* populations, which were more motile than an unpassaged ancestor, were able to better colonize GF larval zebrafish (29), we hypothesized that MR-1 could similarly improve host colonization in our study via enhanced motility. A common assumption in microbiology is that there is a trade-off between adherence and motility (42–45), wherein cells that tend to be more adherent also tend to be less motile and vice versa. Given the difference we observed between the ability of L3 and L5 to adhere to surfaces, we wondered whether these strains might also exhibit a difference in motility.

**FIG 7:**
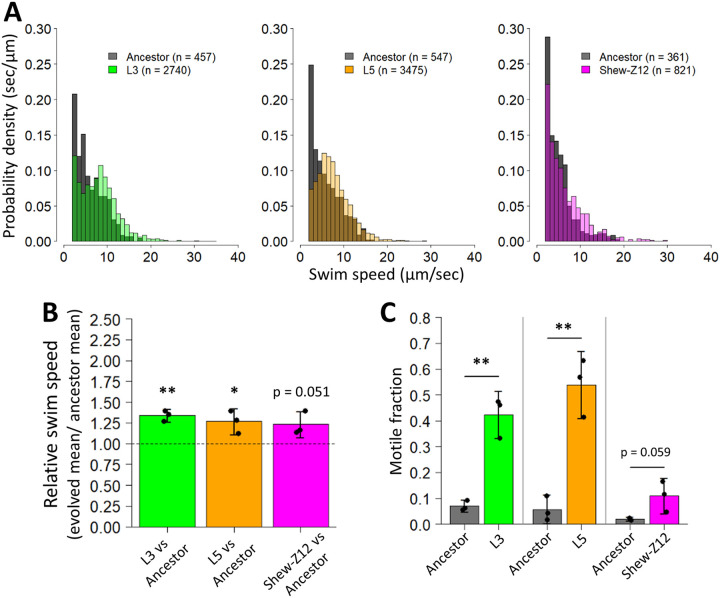
Motility characteristics of evolved and zebrafish isolates compared to ancestor. (A) Histograms comparing the swim speeds of L3, L5, and Shew-Z12 with the ancestral MR-1 strain are shown based on aggregated observations from three flasks per comparison. (B) Mean swim speeds of L3, L5, and Shew-Z12 relative to the ancestor. For each flask, represented by a point, the mean mutant swim speed was divided by the mean ancestral swim speed. One-tailed *t* tests against a μ value of 1 (our null expectation) were conducted to assess whether each group exhibited swim speeds that were significantly faster than the ancestor (*, *P* < 0.05; **, *P* < 0.01). (C) Fractions of L3, L5, Shew-Z12, and ancestral MR-1 populations that were observed to be motile (swimming speeds > 2 μm/s). Each point represents the motile fraction for a single flask, and each bar represents data from three separate flasks. For each panel, the L3 and L5 comparisons were performed under competition, while the Shew-Z12 comparison was performed under monoassociation conditions. Error bars indicate the 95% confidence interval.

We quantified the cellular swimming speeds and motile population fractions of these strains within flasks of GF larvae, by analyzing fluorescence microscopy images with an automated cell tracking algorithm. These assays were conducted in competition (evolved isolate versus ancestor) to mimic the conditions under which we assessed fitness. Thus, if differences in motility were dependent on the competitive dynamics between the evolved isolates and their ancestor, we would capture that in these assays. Surprisingly, despite their differing biofilm phenotypes, we found that both the L3 and L5 isolates were more motile than the ancestor. The evolved strains demonstrated both faster speeds (Fig. 7A and B), and they had a larger portion of their population that was motile (Fig. 7C). These results confirm that motility can be important for larval zebrafish colonization.

We next compared the motility phenotypes of our MR-1 ancestor and evolved isolates to the zebrafish isolate. Our hypothesis was that Shew-Z12 would also exhibit greater motility than the MR-1 ancestor. We examined Shew-Z12 motility as described above, with the exception that Shew-Z12 and our ancestral MR-1 strains were assayed in monoassociation. Attempts to fluorescently tag Shew-Z12 were unsuccessful, leaving no way to distinguish this species via fluorescence microscopy, and thus we collected data for these assays in bright field. We found that Shew-Z12 too exhibited faster swimming speeds and a greater motile fraction of the populations compared to the MR-1 ancestor (Fig. 7). This supports the hypothesis that in our study MR-1 evolved along an adaptive trajectory that is phenotypically convergent with Shew-Z12.

### The MshL missense mutation is sufficient to explain evolved phenotypes.

**FIG 8:**
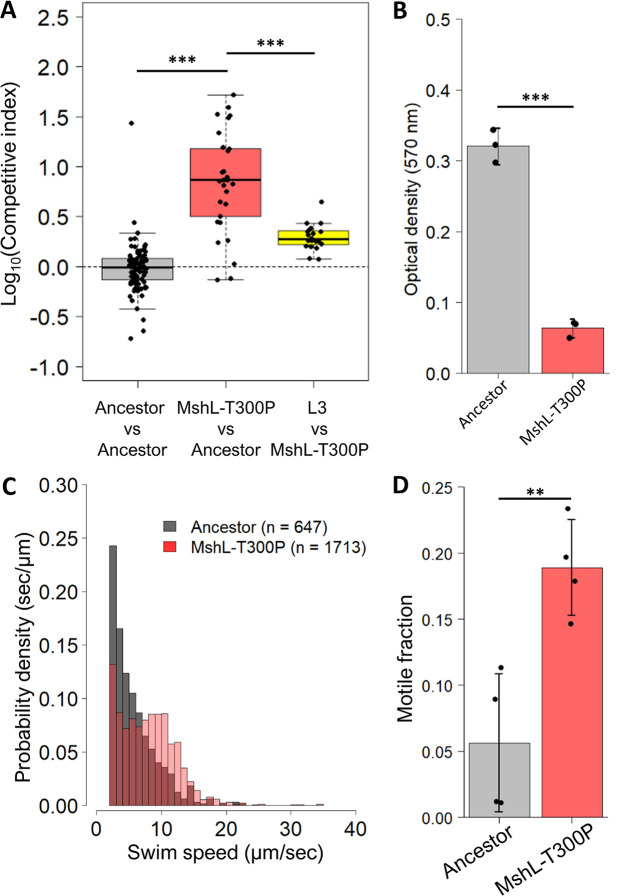
Effects of MshL-T300P on competitive fitness, biofilm formation, and motility. (A) Competitive ability of the MshL-T300P mutant against the ancestral MR-1 reference strain (middle box) and the L3 isolate (right most box). The ancestral control competition (tagged ancestor versus untagged ancestor) is shown in the leftmost box, and these data repeated from Fig. 3B and 4A. A one-tailed *t* test was conducted to determine statistical relationships between group means (***, *P* < 0.001). Each point represents the competitive index measured for a single larval gut culled from at least three replicate flasks (*n* = 89 guts for ancestor versus ancestor, 30 guts for MshL-T300P versus ancestor, and 20 guts for MshL-T300P versus L3). (B) The optical density (570 nm) corresponds to crystal violet intensity. Higher optical density readings indicate more robust biofilms. A one-tailed *t* test was conducted to determine statistical relationships between group means (***, *P* < 0.001). (C) Histograms comparing the swim speeds of the MshL-T300P mutant with the ancestral MR-1 strain are shown based on aggregated observations from four flasks per comparison. MshL-T300P swim speed, 8.2 ± 1.2 μm/s; ancestor swim speed, 5.6 ± 0.5 μm/s (means ± the standard deviations). (D) Fractions of MshL-T300P and ancestral MR-1 populations that were observed to be motile (swimming speeds > 2 μm/s). Each point represents the motile fraction for a single flask, and each bar represents data from four separate flasks. A one-tailed *t* test was conducted to determine statistical relationship between group means (**, *P* < 0.01). For panels B and D, the error bars indicate the 95% confidence interval.

Intriguingly, in a prior study Jones et al. used soft agar motility assays to demonstrate that V. cholerae *mshA* deletion mutants, which lack the major pilin subunit of the Msh pilus, exhibited larger migration zones compared to wild-type V. cholerae (46). Given the increased motility observed in our evolved isolates and the prevalence of *msh* operon mutations in our sequencing data, we tested whether the missense mutation we found in the L3 isolate (MshL-T300P) was sufficient to recapitulate its evolved phenotypes. We constructed this mutation in the ancestral MR-1 genomic background and assessed the competitive fitness, biofilm-forming capacity, and motility phenotypes of the resulting mutant. For all three phenotypes, the MshL-T300P mutant performed similarly to the L3 isolate which contained this mutation (Fig. 8), demonstrating the sufficiency of this mutation to generate the evolved phenotypes. As before, we conducted our motility assays under competitive conditions, using a fluorescently labeled version of the MshL-T300P mutant, to mimic the conditions we used to assess the L3 isolate’s motility. In addition, to assess whether the MshL-T300P mutation was the primary driver of increased fitness in the L3 isolate, we competed the L3 isolate against the MshL-T300P mutant. We hypothesized that if the MshL-T300P mutation imparted an increase in fitness that was comparable to the L3 isolate, competing them should result in a dramatic reduction in the L3 isolate’s competitive index. This is precisely what we observed (Fig. 8A, rightmost column), implying that the MshL-T300P mutation was the primary driver of adaptation in the L3 isolate. Importantly, the fact that the competitive indices measured for the latter competition were all greater than 1 (>0 after log transformation), could indicate the presence of additional adaptive mutations in the L3 isolate.

## DISCUSSION

We serially passaged replicate bacterial populations through the intestines of GF larval zebrafish to explore how a non-host-associated bacterium adapts to a novel vertebrate host. We expected that there would be an abundance of niche space available to evolving MR-1 populations. For example, cells capable of colonizing larval guts must compete externally in the aqueous environment in order to access the larvae, and then compete *in vivo* so they will be sampled and carried over in subsequent passages. Both environments likely contain unique sets of selective pressures. On one hand, we hypothesized that this might select for divergent adaptive trajectories resulting in unique genotypes, and associated phenotypes, that would allow for the exploitation of distinct niches. Alternatively, if selection resulted in adaptation to a single niche within our system, we expected that specialists for that niche would exhibit high relative fitness, leading to phenotypic parallelism among adaptive lineages. Such parallelism could indicate that only a small number of traits are likely to facilitate the initiation of host associations. Moreover, because traits that are common among independent adaptive genotypes have an increased likelihood of playing a causal role in enhancing fitness, such parallelism could provide evidence that phenotypes are adaptive. To address these hypotheses, we examined the mutations and phenotypes of two evolved isolates with the highest mean fitness in our experimental system: isolates from the L3 and L5 populations.

Interestingly, the improved competitive fitness we observed in the L3 and L5 isolates was associated with unique genotypes (see Table S1), and this suggested that each isolate found idiosyncratic strategies for adaptation. L3 contained a missense mutation in the *mshL* gene, and mutations in the *msh* operon were observed in five of six of the evolved isolates we sequenced (Fig. 4A, lower grid; see also Table S1). Further, we demonstrated the MshL-T300P mutation found in the L3 isolate was sufficient to significantly improve larval colonization, which supports the conclusion that the *msh* operon mutations we observed were likely adaptive. The Msh pilus has also been implicated in several other host-microbe systems (12, 33–36), and therefore focusing on the products encoded by this operon may yield fruitful avenues for researchers interested in manipulating host-microbe interactions.

Less clear is which mutations in the L5 isolate’s genome are adaptive. The mutations we identified fell within genes that were annotated as a sensor histidine kinase, a lipoprotein, a diguanylate cyclase, and a hypothetical protein (see Table S1). These mutations could affect a broad range of cellular and physiological processes. Intriguingly, several studies have shown that diguanylate cyclase expression can affect biofilm and motility behaviors in Gram-negative bacteria (46–50); however, without further investigation it is difficult to predict which mutations observed in the L5 isolate actually play an adaptive role in MR-1’s ability to colonize larvae. We plan to disentangle the effect of these mutations in future work.

Regarding the biofilm phenotypes we assessed, the L3 isolate displayed a reduced ability to form biofilms, while neither the L5 isolate nor Shew-Z12 exhibited biofilms that differed significantly from the MR-1 ancestor phenotype. Although this suggests biofilm formation does not correlate with competitive fitness, the cell densities required to generate detectable biofilms in these assays are orders of magnitude higher than would be experienced by MR-1 in a larval flask. Therefore, it is possible the aggregative behaviors we observed are not representative of the behaviors MR-1 isolates would manifest when competing to colonize the larval gut. Nonetheless, the ability to form robust biofilms could pose several advantages with respect to host colonization. Bacteria are known to form intimate associations with host epithelial tissues, and the ability to form biofilms can be critical to successful host colonization and host specificity (51, 52). In addition, bacterial aggregates can shield internal members from harsh environmental conditions until more favorable conditions are encountered within a host, wherein large numbers of cells can detach and colonize (53). Similarly, upon colonization of a host, biofilms can also protect bacterial members from harmful host defenses (54). Given these potential advantages, it makes sense that the evolved isolate with the highest mean fitness, L5, would maintain a robust ability to form biofilms.

Conversely, we demonstrated that the L3 isolate’s reduced biofilm phenotype could be explained by the MshL-T300P mutation. *MshL* encodes a putative outer membrane pore protein through which the Msh pilus extends (32, 46, 55, 56). Consistent with what others have found for the Msh pilus, if the MshL-T300P mutation was a loss of function mutation, it could reduce MR-1’s Msh pilus expression—and its ability to form biofilms—resulting in a more planktonic existence (46, 57–59). In turn, this could increase L3’s encounter rate with zebrafish larvae that are swimming through the aqueous environment. In this way, the L3 isolate would have a competitive advantage over ancestral cells that were adherent to flask surfaces and were therefore less able to access larval hosts. We found some support for this mode of adaptation in that evolved isolates L2-L6 appear to outcompete the ancestor in the aqueous environment outside the larvae during our competitive fitness assays (Fig. S3). However, the CFU counts used in these assessments occurred under conditions that permitted population exchanges between larval guts and the external environment. If evolved MR-1 strains had a distinct *in vivo* advantage, efflux from the larval digestive tract could inflate external counts, making it difficult to disentangle the influence of each portion of the experimental environment on competitive fitness. We plan to dissect the impacts of different features of our system on the fitness of evolved isolates in a future work. Alternatively, reduced expression of the Msh pilus could potentially help the L3 isolate evade elements of the larval immune system upon gut colonization (12, 36). Neither of these hypotheses necessarily accounts for the L3 isolate’s faster swimming speeds, although speculatively, it is possible that if MR-1 were not producing pili, it might be able to devote more resources to motility which is energetically costly (60).

10.1128/mBio.01519-20.3 FIG S3 Competitive outcome in embryonic medium during fitness competitions. (A) Relative performance of evolved MR-1 isolates compared to the ancestral strain in the aqueous environment outside larvae during colonization competitions. The data from an ancestral competition between tagged and untagged ancestral strains (leftmost box) is shown as a control. Each point represents the competitive index measured for a separate replicate flask (*n* = 6 flasks for the ancestor versus ancestor control, and three flasks for all other groups). Error bars indicate the 95% confidence intervals for each group’s mean. Download FIG S3, TIF file, 0.2 MB. Copyright © 2020 Lebov et al. 2020 Lebov et al. This content is distributed under the terms of the Creative Commons Attribution 4.0 International license.

In our study, the fact that the L3 and L5 isolates evolved to outcompete the ancestor, while exhibiting distinct biofilm phenotypes, suggests that each isolate could be pursuing alternative adaptive strategies. However, even though biofilm formation often trades off with motility (43–45), we observed selection for enhanced motility via two separate genetic pathways, which lends support to the conclusion that augmented motility is adaptive. Further, this phenotype was also observed in the closely related zebrafish isolate, Shew-Z12. Interestingly, the motile fraction of Shew-Z12’s population is reduced compared to either the L3 or L5 isolates (Fig. 7C). This could stem from the fact that Shew-Z12 evolved its motility characteristics in the presence of a bacterial community, whereas our experiment was conducted under germfree (i.e., axenic) conditions. It is possible that this historical difference may have altered the costs or benefits of motility, leading Shew-Z12 to evolve distinct motility characteristics. For example, cross-feeding between community members could alter bacterial foraging requirements and result in unique motility optimization (61, 62).

Although it is not clear exactly how motility improves host colonization in our system, one possibility is that enhanced motility increases chemotactic responses to host-produced chemical gradients, thereby increasing bacterial encounter rates with larval zebrafish. Alternatively, once an evolved MR-1 cell encounters a host, faster swimming speeds could help strains traverse narrow junctions within the host to reach the gut more quickly than their ancestral competitor (14). However, prior work showed no fitness advantage for a hypermotile *Aeromonas* strain gavaged into the oral cavity of larval zebrafish, suggesting this mode of fitness enhancement may be less likely (29). Another option is that after bacterial cells migrate into the digestive tract, motility could help bacteria resist expulsion induced by intestinal contractions (39). Ultimately, our results add to a growing body of evidence implicating motility as an important trait for host colonization (14, 17, 29, 63), and the phenotypic parallelism we observed suggests that traits associated with dispersal can play a critical role in the establishment of host-microbe symbioses (64).

## MATERIALS AND METHODS

### Zebrafish husbandry.

To ensure animal specimens were treated ethically in all experiments involving zebrafish, we adhered to the standard protocols and procedures approved by the University of Oregon Institutional Animal Care and Use Committee (IACUC protocol 15-98). GF derivations were carried out as described by Melancon et al. (70). Details about larval gut dissections can be found the Serial passage section.

### Bacterial strains.

Our ancestral reference S. oneidensis (MR-1) and Shew-Z12 strains were obtained from Karen Guillemin’s laboratory at the University of Oregon. Detailed protocols for all modifications to MR-1, including Tn*7*-mediated gfp and dTomato insertions as well as allelic exchange applications, can be found in Wiles et al. (65). The MshL-T300P mutation allelic construct was created via PCR amplification of the mutated segment from the evolved L3 isolate’s genome, and the resulting mutant (MshL-T300P) genetically differs from the ancestor by only a single base pair. All S. oneidensis strains were cultured in tryptic soy broth at 30°C under shaking conditions.

### Serial passage.

Overnight tryptic soy broth (TSB) cultures (5 ml) of MR-1 isolates tagged with either green fluorescent protein (MR-1gfp) or dTomato fluorescent protein (MR-1dT) were diluted 1:100 in TSB and allowed to grow out to late log phase (4 to 5 h). Six replicate ancestral populations were then generated by combining subcultures of MR-1gfp and MR-1dT at a 1:1 ratio. These mixtures allowed us to infer the occurrence of adaptive events based on fluorescent tag frequency changes observed throughout the experiment (see Fig. S2). Beneficial mutations occurring in a tagged genomic background should cause the frequency of that tag to increase over time. Next, 10 μl of each of these replicate ancestral populations were used to inoculate larval flasks containing ∼15 ml of embryonic medium and ∼15 GF larval zebrafish at 4 days postfertilization (dpf; inoculating MR-1 densities were ∼10^6^ CFU/ml). Larvae were then incubated with MR-1 populations at 28°C for 72 h. At 7 dpf, 10 larvae were euthanized with tricane and mounted on a glass slide, and their digestive tracts were dissected. Glass slides were coated with 3% methylcellulose to help immobilize larvae during dissections. After the dissections, all 10 digestive tracts culled from each flask were placed in a single 1.7-ml tube containing 500 μl of EM and ∼100 μl of 0.5-mm zirconium oxide beads (Next Advance, Averill Park, NY). The contents the larval guts in each of these tubes were then immediately homogenized using a bullet blender tissue homogenizer (Next Advance) for 60 s at power 4. To preserve our ability to revive replicate populations after each passage, we created freezer stocks by using a pipette to mix 200 μl from each homogenized tube with 200 μl of 50% glycerol (25% glycerol final concentration). These freezer stocks were stored at −80°C. The remaining contents of each homogenized tube was then stored at 4°C for 0 to 14 days, at which point ∼250 μl were sampled to inoculate a subsequent set of GF larval flasks (∼15 larvae in ∼15 ml of EM). Upon inoculation, 100 μl of each larval flask was dilution-plated in triplicate to quantify the inoculating population densities (typically ∼10^3^ CFU/ml) and determine tag frequencies. This cycle was repeated for 20 passages. All six replicate evolving populations were maintained separately throughout our experiment.

### Comparative genomics.

We submitted our MR-1 and Shew-Z12 strains to the Washington State University Molecular Biology and Genomics Core (WSUGC) for long read sequencing. Genome assembly for MR-1 was conducted by WSUGC, whereas genome assembly for Shew-Z12 was conducted in-house with Canu v1.7.1 (66). To generate annotation files for these genomes, we relied on Prokka v1.12 (67), and RAST v2.0 (68).

### Phylogenetics.

Using Integrated Microbial Genomes and Microbiomes (69) (IMG/M; https://img.jgi.doe.gov/), we collated a set of 16S rRNA genes from 28 *Shewanella* species, 2 *Vibrio* species, and 1 *Aeromonas* species (see Table S2 for metadata). These 16S rRNA genes were entered into Clustal Omega (https://www.ebi.ac.uk/Tools/msa/clustalo/) to generate a multiple sequence alignment file and a subsequent Newick-formatted phylogenetic tree file. This file was then visualized with FigTree v1.4.4 (http://tree.bio.ed.ac.uk/software/figtree/).

### Genome comparisons between *S. oneidensis* and other *Shewanella* species.

The average sequence identity (ANI) was calculated using the EZBioCloud online ANI calculator (71) to quantify the ANI between S. oneidensis and Shew-Z12.

### Specific gene and operon comparisons between *S. oneidensis* and Shew-Z12.

ANI was calculated using the same tool described in our whole-genome comparisons above. For MshH-Q comparisons, we separately concatenated the amino acid sequences of each gene in the *mshH-Q* operons of S. oneidensis and Shew-Z12 in series by relying on our RAST-annotated files. We then generated multiple sequence alignment (msa) files using Clustal Omega web tool (72) that compared these MshH-Q sequences and used them to depict sites of divergence along the *mshH-Q* operon (Fig. 5). Visualizations of single gene or multigene comparisons between S. oneidensis and Shew-Z12 were created using Clustal Omega-based msa files that were imported into Jalview2 (73). To highlight regions of divergence within genes, we color-coded our comparisons using the color by annotation feature of Jalview2 (Fig. 5). This feature color codes amino acid comparisons per site based on biochemical conservation.

### Evolved mutation calling.

We selected one randomly chosen isolate per evolved replicate population (six isolates total) by using an inoculation loop to dilution streak a sample from each population’s freezer stock on TSA (tryptic soy agar) plates (one plate per evolved population, totaling six plates) and incubating the plates at 30°C for 24 h. For each population, we overnight cultured four colonies that resulted after 24 h of growth and then created freezer stocks (stored at –80°C) that consisted of a 1:1 mixture of each cultured isolate and an equal volume of 50% glycerol (final concentration, 25% glycerol). To create genomic libraries for each evolved isolate, we used an inoculation loop to generate overnight cultures from each isolate’s corresponding freezer stock and then extracted genomic DNA from each culture using a Promega Wizard genomic DNA purification kit (catalog no. A1120). Single-end 150-bp libraries were generated from these genomic DNA extractions according to the Nextera XT DNA library prep kit reference guide (Document 15031942 v02), and these libraries were sequenced on the Illumina HiSeq 4000. According to this same protocol, we also sequenced the genomes of our ancestral MR-1gfp and MR-1dT strains on the Illumina HiSeq 4000. To identify candidate adaptive mutations, we used breseq 0.31.0 in consensus mode to compare each Illumina sequenced isolate to the annotated ancestral reference separately and then looked for single nucleotide polymorphisms and indels that were present in each evolved isolate but absent in MR-1gfp and MR-1dT isolates (74). With the exception of *mshH-Q* genes, the gene annotations for the mutations listed in Table S1 were determined by Prokka v1.12 (67). The *mshH-Q* gene annotations were determined by RAST v2.0.

### Competition assays.

Overnight 5-ml TSB cultures of competing strains were diluted 1:100 in TSB and allowed to grow to late log stage (4 to 5 h). Then, 500 μl of each competitor was then mixed in a single 1.7-ml tube so that competitors were at an approximate 1:1 ratio. Competition mixtures were pelleted (7,000 relative centrifugal force [rcf] for 5 min) and resuspended in 1 ml of sterile EM. Resuspended competition mixtures were diluted 1:100, and 7.5-μl portions of these dilutions were used to inoculate GF larval flasks containing ∼15 ml of EM and ∼15 larvae at 4 dpf. Immediately after inoculation, triplicate 100-μl samples from each competition flask were dilution plated to establish the inoculation ratio of competitor 1 to competitor 2 (CFU/ml). After inoculation, flasks were incubated at 28°C for 72 h. At 7 dpf, embryonic medium samples were taken from each flask to quantify CFU in the water column, and multiple larvae per flask were euthanized with tricaine (75). Their guts were dissected and individually placed in 1.7-ml tubes containing 500 μl of sterile EM and ∼100 μl of 0.5-mm zirconium oxide beads (Next Advance). The contents the larval guts in each these tubes were then immediately homogenized using a bullet blender tissue homogenizer (Next Advance) for 60 s at power 4. Homogenized tubes were then dilution plated to determine the CFU/gut for each competitor. A competitive index (CI) was calculated for each dissected gut by dividing the ratio of competitor 1 to competitor 2 found in each gut by the mean inoculation ratio determined from the triplicate measurements for each corresponding flask, as follows:

$$\text{CI = }\frac{{\text{competitor 1:competitor 2}}_{\text{guts}}}{{\text{competitor 1:competitor 2}}_{\text{inoculum}}}.$$

We used Dunnett contrasts to compare dissected larval guts grouped from separate flasks with a linear model where flasks were considered a random effect.

### Rich medium competitions.

Overnight TSB cultures of competing strains were diluted 1:100 in TSB and allowed to grow to late log stage (4 to 5 h). Then, 500 μl of each competitor was then mixed in a single 1.7-ml tube so that competitors were at an approximate 1:1 ratio. Competition mixtures were pelleted (7,000 rcf for 5 min) and resuspended in 1 ml of TSB. Resuspended competition mixtures were diluted 1:100, and 5-μl portions of these dilutions were added to 10 ml of TSB in a 20-ml test tube. Immediately after inoculation, triplicate 100-μl samples from each competition culture tube were dilution plated to establish the inoculation ratio of competitor 1 to competitor 2 (CFU/ml). We incubated each competition at 30°C for 24 h with agitation (angled back and forth rocker, 60 rpm), at which point we again took triplicate 100-μl samples from each competition tube and dilution plated them to quantify the CFU/ml for each competitor. Competitive indices were calculated by dividing the final CFU ratio of competitor 1 to competitor 2 by the inoculation ratio, as follows:

$$\text{CI = }\frac{{\text{competitor 1:competitor 2}}_{\text{final}}}{{\text{competitor 1:competitor 2}}_{\text{inoculum}}}.$$

### Biofilm assay.

Triplicate biological replicate overnight TSB cultures of strains of interest were diluted 1:100 in TSB and allowed to grow out to late log stage (3 to 5 h). Next, 1-ml portions of subcultured strains were pelleted (7,000 rcf for 5 min), and the pellets were normalized to an optical density at 600 nm of 1.0 via resuspension with sterile EM. Then, 150-μl portions of each resuspension were added to four wells of a round-bottom 96-well polystyrene plate (Greiner Bio-One, catalogue no. 650185) per biological replicate. The plate was incubated at 30°C for 24 h, and the volume of each well was removed with a multichannel pipette. The wells were rinsed twice with 200 μl of sterile EM, and each well was stained with 180 μl of 0.1% crystal violet (CV). The plate was incubated at room temperature for 10 min, at which point the crystal violet was removed with a multichannel pipette, and the wells were again rinsed twice with 200 μl of sterile EM. The CV was solubilized with 100% dimethyl sulfoxide (DMSO) for 15 min with agitation (∼180 rpm on a rotating minishaker), and 100 μl of the solubilized CV was added to 100 μl of 100% DMSO in a flat-bottom 96-well polystyrene plate (Corning, Inc., reference no. 3595). The optical densities were then measured for each well at 570 nm.

### Motility assays.

Next, 5-ml overnight TSB cultures of strains of interest were diluted 1:100 in TSB and allowed to grow to late log stage (4 to 5 h). Strains were then prepped for inoculation as described above. A volume of 7.5 μl of each strain prepared for inoculation was used to inoculate GF larval flasks containing ∼15 ml of EM and ∼15 larvae at 4 dpf. Inoculated larval flasks were incubated at 28°C for 13 to 17 h, at which point bacteria in each flask were imaged on an inverted microscope (Nikon Eclipse Ti-e) by focusing on the bottom interior surface of the flask. Ten 30- to 45-s movies were then captured at a rate of 15 to 24 frames/s. When movies were taken of competing populations, each population was fluorescently tagged with either gfp or dTomato, and movies were taken separately to capture the motility dynamics of each tagged population independently within the same flask. Single strain movies were taken in a bright field. Bacteria tracking was performed in MATLAB using previously described software (https://pages.uoregon.edu/raghu/particle_tracking.html). In brief, bacterium-like objects were identified by intensity thresholding after bandpass filtering and then localized using a radial symmetry algorithm (76). Tracks were assembled using nearest-neighbor linking. Tracks shorter than five frames were discarded. As an additional filtering step to remove multiple tracks assigned to the same, nonmotile bacterium, tracks with a difference in mean position of <1.7 μm were culled, keeping only the longest track. Based on the measured distribution of swimming speeds, a cutoff of 2 μm/s was used to operationally distinguish a “motile” bacterium from a “nonmotile” one.

### Data availability.

Our genome assemblies and raw sequencing files are available for download under BioProject accession no. PRJNA633711.
