# Supplementary figures and images for: Phenotypic Parallelism during Experimental Adaptation of a Free-Living Bacterium to the Zebrafish Gut
Source: mBio. 2020 Aug 18;11(4):e01519-20. doi: 10.1128/mBio.01519-20 (PMC7439477; doi:10.1128/mBio.01519-20)

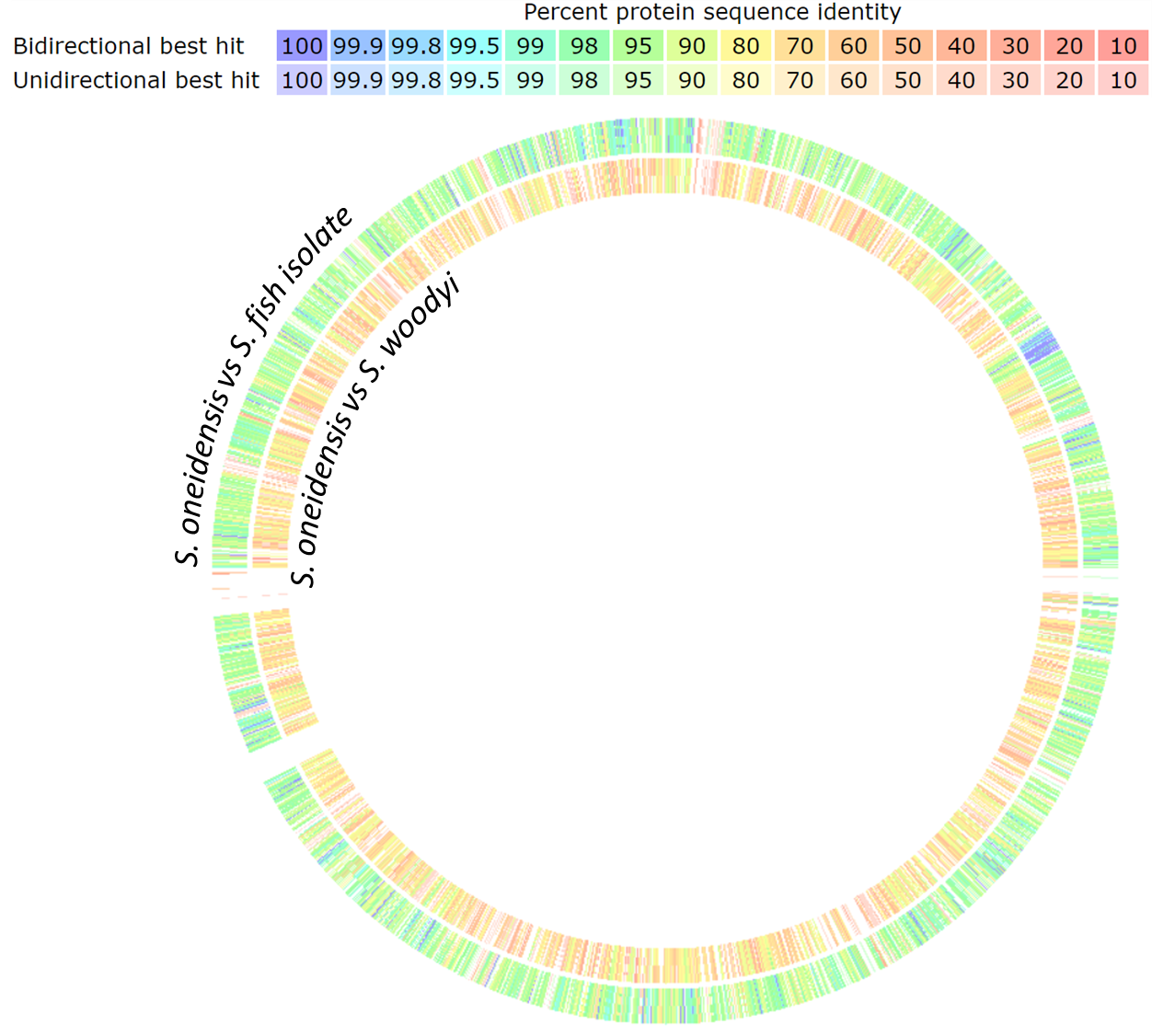

Supplement: FIG S1 [file mBio.01519-20-sf001.tif]

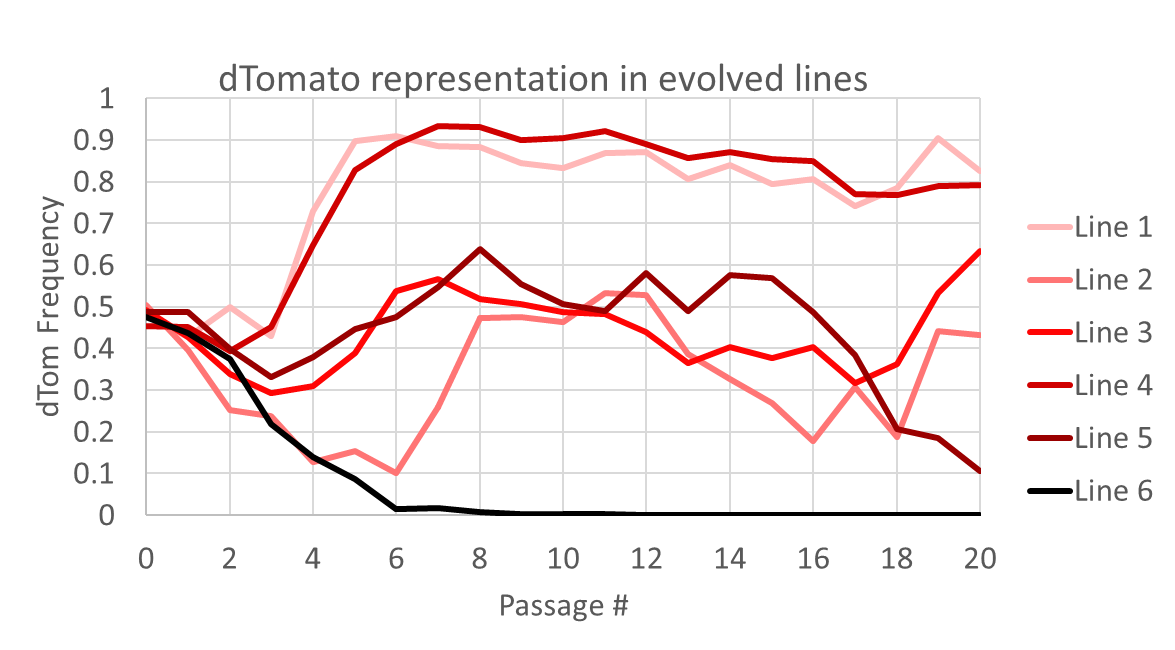

Supplement: FIG S2 [file mBio.01519-20-sf002.tif]

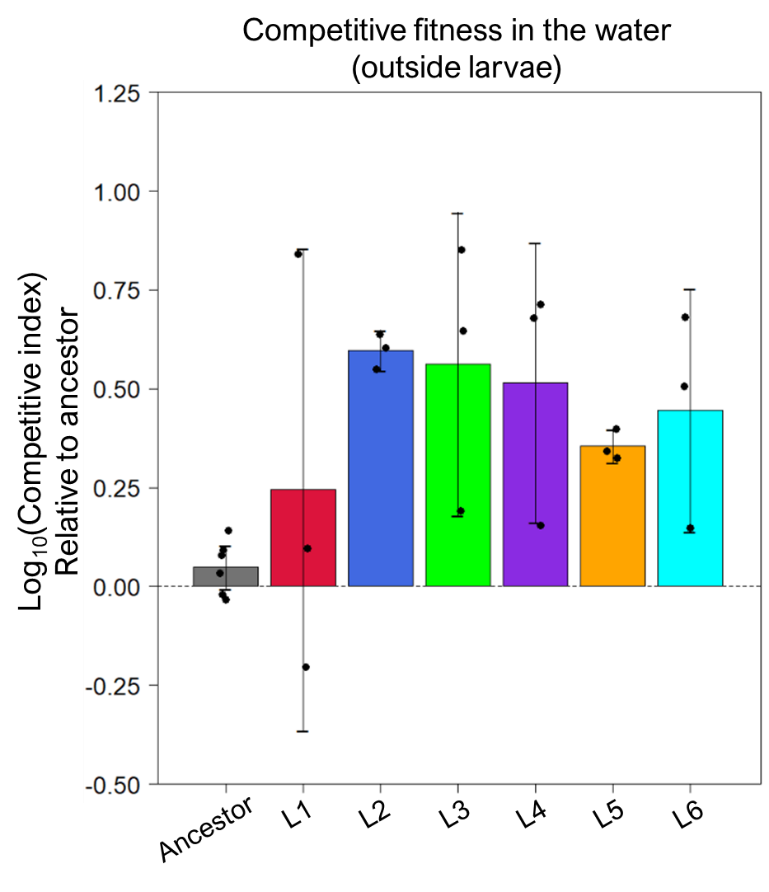

Supplement: FIG S3 [file mBio.01519-20-sf003.tif]
